# Academic performance in the oral surgery subjects of undergraduate dental students: a cross-sectional study

**DOI:** 10.21142/2523-2754-1401-2026-271

**Published:** 2025-12-28

**Authors:** José Wittor De Macedo Santos, Otacílio Luiz Chagas, Roberto Pereira Pimentel, Maísa Casarin, Francisco Wilker Mustafa Gomes Muniz

**Affiliations:** 1 Graduation Program in Dentistry, Federal University of Pelotas. Pelotas, Rio Grande do Sul, Brazil. josewittor@hotmail.com Universidade Federal de Pelotas Graduation Program in Dentistry Federal University of Pelotas Pelotas, Rio Grande do Sul Brazil josewittor@hotmail.com; 2 Department of Surgery, Traumatology and Oral and Maxillofacial Prosthesis, Federal University of Pelotas. Pelotas, Rio Grande do Sul, Brazil. otaciliochagasjr@gmail.com Universidade Federal de Pelotas Department of Surgery Traumatology and Oral and Maxillofacial Prosthesis Federal University of Pelotas Pelotas, Rio Grande do Sul Brazil otaciliochagasjr@gmail.com; 3 Departament of Periodontology, Federal University of Pelotas. Pelotas, Rio Grande do Sul, Brazil. pimentelrobp@gmail.com maisa.66@hotmail.com wilkermustafa@gmail.com Universidade Federal de Pelotas Departament of Periodontology Federal University of Pelotas Pelotas, Rio Grande do Sul Brazil pimentelrobp@gmail.com maisa.66@hotmail.com wilkermustafa@gmail.com

**Keywords:** academic performance, academic success, education, illicit drugs, rendimiento académico, éxito académico, educación, drogas ilícitas

## Abstract

**Objective:**

This study assessed the factors influencing the academic performance of undergraduate dental students in the field of oral surgery at a public university in southern Brazil.

**Materials and Methods:**

It included 146 students who were regularly enrolled in an undergraduate dental program, who completed at least one oral surgery subject. The survey questionnaire encompassed various variables, including sex, age, sexual orientation, family income, ethnicity, part-time employment, use of substance (licit or illicit), and whether dentistry was their initial choice for an undergraduate course. Academic performance was gauged using a grading system ranging from 0.0 (lowest possible grade) to 10.0 (highest possible grade). Bi- and multivariable linear regression analyses were performed to examine the associations between the outcome and independent variables.

**Results:**

An inverse relationship between age and academic performance remained significant (β: -0.318; 95% confidence interval [95%CI]: (-0.101--0.034). Female students outperformed male students (β:0.173; 95%CI:0.022-0.400), while students who reported an increase in drug use exhibited lower academic performance (β: -0.158; 95% CI: -0.354--0.001).

**Conclusions:**

It was concluded that academic performance in oral surgery subjects was notably poorer in older students and those who reported increased drug use. Female students, on the other hand, demonstrated better academic performance.

## RESUMEN

**Objetivo:**

Este estudio evaluó los factores que influyen en el rendimiento académico de estudiantes de odontología de pregrado en el área de cirugía oral en una universidad pública del sur de Brasil.

**Materiales y métodos:**

: Se incluyó a 146 estudiantes matriculados regularmente en un programa de odontología de pregrado, que cursaron al menos una asignatura de cirugía oral. El cuestionario abarcó diversas variables, como sexo, edad, orientación sexual, ingresos familiares, etnia, empleo a tiempo parcial, consumo de sustancias (lícitas o ilícitas) y si la odontología fue su primera opción de carrera de pregrado. El rendimiento académico se midió mediante un sistema de calificación que va de 0,0 (la calificación más baja posible) a 10,0 (la calificación más alta posible). Se realizaron análisis de regresión lineal bivariable y multivariable para examinar las asociaciones entre el resultado y las variables independientes.

**Resultados:**

Una relación inversa entre la edad y el rendimiento académico se mantuvo significativa (β: -0,318; intervalo de confianza del 95% [IC 95%]: -0,101 a -0,034). Las estudiantes mujeres superaron a los estudiantes hombres (β: 0,173; IC95%:0,022-0,400), mientras que los estudiantes que reportaron un aumento en el consumo de drogas exhibieron un rendimiento académico más pobre (β:-0,158; IC 95%: -0,354 a -0,001).

**Conclusiones:**

Se concluyó que el rendimiento académico en las asignaturas de cirugía oral fue notablemente más pobre en los estudiantes mayores y en los que reportaron un mayor consumo de drogas. Las estudiantes mujeres, por otro lado, demostraron un mejor rendimiento académico.

## INTRODUCTION

Academic performance is a multifaceted phenomenon influenced by a range of demographic, socioeconomic, psychological, and personal factors. Moreover, it is essential to factor in institutional elements, including faculty qualifications and teaching-learning methodologies, as they can significantly impact academic performance ^1^. Recognizing its linkage to students' economic well-being, physical health, and mental health further underscores its significance ^2^^,^^3^. Individuals who juggle work alongside their undergraduate studies often contend with demanding schedules, leading to overexertion and potentially resulting in lower academic performance ^4^.

It is crucial to place special emphasis on the well-established reciprocal relationship between mental health and academic performance, as documented in the literature ^5^. Dental students frequently encounter stress as part of their training. A recent systematic review comprising 124 studies found that when evaluating the impact of stress on dental students, academic performance was the most commonly assessed aspect. Alarmingly, nearly half of these studies reported adverse effects of stress on academic performance ^6^.

Within oral surgery subjects, dental students often work with patients who are grappling with physical and emotional distress due to pain, potential traumatic injuries, and aesthetic and functional issues stemming from dental extractions. Additionally, these patients frequently experience extreme anxiety about the upcoming procedures. Surprisingly, there has not been a study assessing the academic performance of undergraduate dental students in oral surgery subjects and the factors that may be associated with it. Nevertheless, existing literature does indicate that maxillofacial surgeons and residents contend with high levels of occupational stress ^7^. 

Therefore, this study aimed to examine the factors linked to academic performance in oral surgery subjects among undergraduate dental students at a public university in southern Brazil. The working hypothesis was that students with lower family income, non-white ethnicity, engaged in part-time employment, and using licit or illicit drugs would demonstrate notably lower academic performance in these subjects.

## MATERIALS AND METHODS

### Study Design

The population for this cross-sectional study encompassed all undergraduate dental students who completed at least one oral surgery subjects at a public university situated in Pelotas, RS, Brazil. The study adhered to the Strengthening the Reporting of Observational Studies in Epidemiology (STROBE) guidelines. The study received ethical approval from the local Human Research Ethics Committee (protocol #3910723 - at March 11^th^ 2020).

### Sample, Inclusion and Exclusion Criteria 

Initially, this is a single institution survey where all students who were actively enrolled in the undergraduate dental program at the School of Dentistry, in March 2020 (totaling n=474), were considered eligible and invited to take part in the study, dispensing a sample size calculation. To confirm students' institutional affiliation (enrollment), verification was conducted with the School of Dentistry's dean. Students who responded to the questionnaire, but did not grant access to their academic records were excluded from the study. Additionally, students who had not completed at least one oral surgery subject during their undergraduate course were also excluded. No further exclusion criteria were applied.

### Data Collection and Independent Variables

Data collection involved the use of a semi-structured questionnaire, which was distributed to students through several institutional communication platforms. Students were contacted through personal emails and liaised with class representatives. Professors and dental students jointly extended the invitation to participate in the study, and access to the questionnaire was provided through their social media profiles.

Due to the societal impacts of the COVID-19 pandemic, the first academic semester of 2020 occurred up to March of that year. Subsequently, academic activities were either postponed or transitioned to remote modes. Data collection took place between June and August 2020. The survey questionnaire, encompassing the variables of interest (such as sex, age, sexual orientation, family income, ethnicity, part-time employment, use of licit or illicit substances, and whether dentistry was their primary choice of undergraduate course), was hosted on the Google Forms platform. It also included a statement allowing access to academic records. For reference, since this was a secondary analysis of a larger study, the complete questionnaire with all the studied variables can be found elsewhere ^8^. The English version for the questions included in the present study are available in Table 1.

**Table 1:** English version for the questions used in the present study for data collection (2020)

Question	Possible answers
1. What is your sex?	Male
	Female
	Other:
2. How old are you (in years)?	________________
3. What is your sexual orientation?	Heterosexual
	Bisexual
	Homosexual
	Other: ___________________
4. In Brazilian Reais, what is your family income?	____________________
5. What is your race?
	White
	Brown
	Black
	Yellow
	Indigenous
	I do not know
6. Was the Dentistry course your first choice for graduation?	Yes
	No
7. Do you have any of the below paid activities (academic or not), and if so, which ones?	No
	Monitoring (teaching scholarship)
	Extension (extension scholarship)
	Research (scientific initiation scholarship)
	Internship (paid internship)
	PET (Portuguese acronym for Programa de Educação tutorial)
	Autonomous
	Civil servant
	Other: ___________________
8. Do you/have you ever used anxiolytic and/or antidepressant medication?	Yes
	No
9. In the last 30 days, have you used alcohol?	Yes
	No
10. In the last 30 days, have you smoked cigarette?	Yes
	No
11. In the last 30 days, have you used any illicit drugs, including marijuana, or any other drug?	Yes
	No

### Outcome

The dependent variable (outcome) in this study was the academic performance in oral surgery subjects. The curriculum of the included School of Dentistry provides three mandatory oral surgery subjects throughout the undergraduate course. The first oral surgery subject is offered to dental students in their fifth semester, and the third one is for those in the seventh semester. The outcome was defined as the average grade obtained across all completed oral surgery subjects. If a student completed only one subject, their grade in that subject was used as the outcome measure.

Academic performance for undergraduate dental students was assessed using a grading scale that ranged from 0.0 (representing the lowest possible grade) to 10.0 (representing the highest possible grade). The data exhibited a parametric distribution, as confirmed by the Shapiro-Wilk test (p=0.468). It is worth noting that access to this data was limited solely to the lead researcher of this study.

### Independent Variables

The following independent variables were considered: sex (male or female), age (in completed years), and skin color (categorized as white and non-white, following the criteria set by the Brazilian Institute of Geography and Statistics, which ethnically classifies the Brazilian population into white, brown, black, yellow, or Indigenous individuals) ^9^. All individuals who self-identified as a skin color other than white were categorized as non-white. It is important to note that in this study, there were no students who identified as yellow or Indigenous.

Sexual orientation (heterosexual and others), and monthly family income (in Brazilian reais) were also collected. Another variable identified whether students had any remunerated activities (dichotomized as yes [formal employment or any scholarship] or no). Students with employment, remunerated internships, or any academic scholarships were classified as participants who performed part-time employment. 

The variable concerning the use of licit or illicit drugs distinguished between students who had used alcohol, tobacco, or marijuana in the past 30 days and those who had not. An objective question also inquired whether dentistry was the students' initial choice for their undergraduate course, with possible responses being "yes" or "no." It is important to note that missing data were only observed in the monthly family income variable, and no efforts were made to impute this missing data.

### Statistical Analysis

All statistical analyses were conducted using SPSS version 29.0 for Mac. Descriptive data analysis involved calculating means and standard deviations (SD) for continuous variables, while categorical variables were summarized using frequency distributions. Given the parametric distribution observed in the primary outcome, bivariable and multivariable linear regression analyses were conducted to examine the associations between the outcome and independent variables.

In the bivariable analysis, variables with p-values < 0.25 were initially included in the multivariable model. However, the use of licit or illicit drugs was included in the final multivariable model regardless of its p-value. The literature indicates a significant impact of this variable on the academic performance of university students ^5^. The final multivariable model was constructed using a "forward" strategy, considering both statistical significance and potential effect modifications within the model. Homoscedasticity was detected, but no multicollinearity was observed. Additionally, residuals were tested, and a symmetrical distribution was also observed. Statistical significance was established at p < 0.05 as the threshold.

## RESULTS

**Figure 1:**
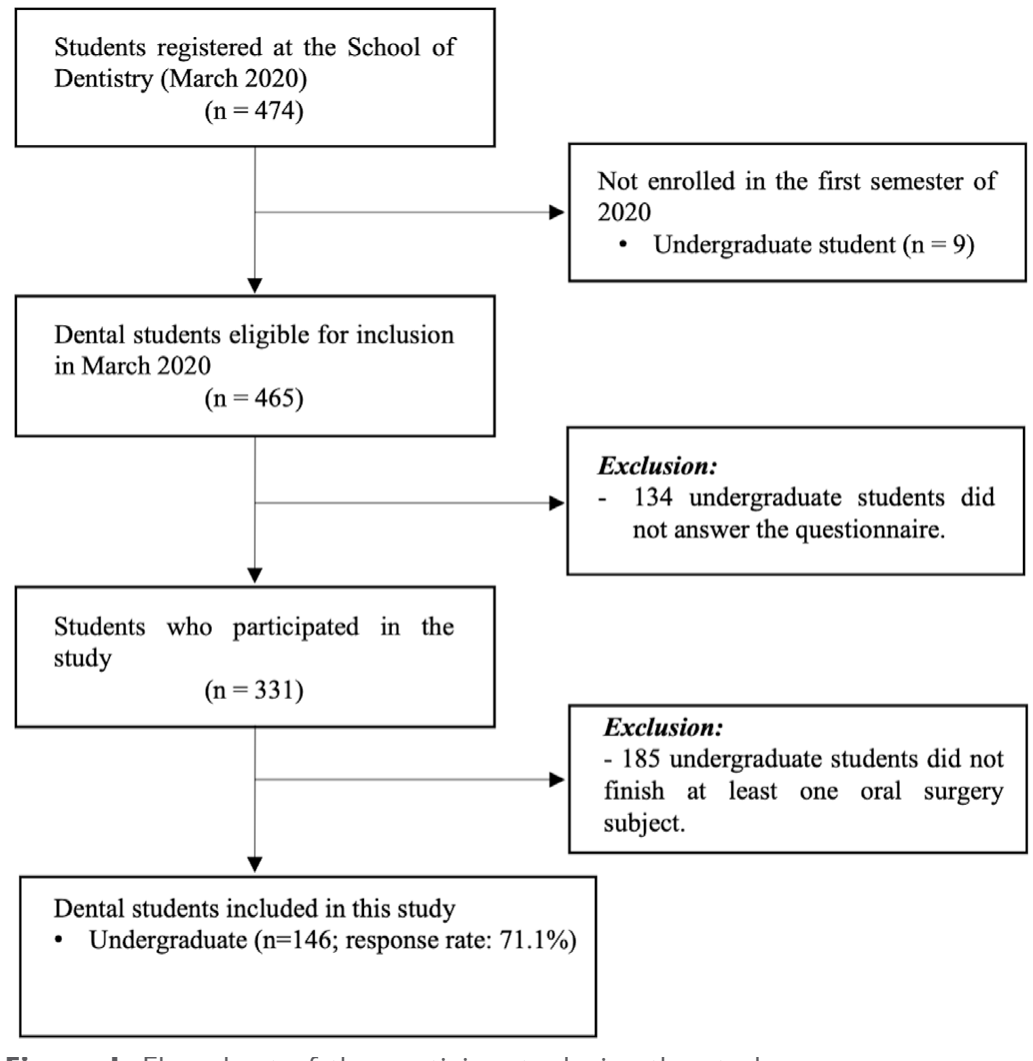
Flowchart of the participants during the study.

In total, 465 undergraduate dental students were enrolled in the undergraduate dental course at the included School of Dentistry in March 2020. Of these, 331 answered the questionnaire (response rate: 71.1%). One hundred and eighty-five students were not included in the final sample of this secondary analysis, as they did not provide access to their academic records (n = 60) or did not complete at least one oral surgery subject yet (n = 125). Finally, 146 dental students were enrolled in oral surgery subjects and comprised the final sample of this study (Figure 1). When considering only those who completed at least one oral surgery subject during data collection, the response rate was 65.2%.

The mean age was 23.39 (SD: 2.63) years. More than half of the sample was composed of females (69.9%) individuals, with white skin color (85.6%), and reported being heterosexual (87%). Brazilian reais mean monthly family income was 8200.00 BRL (approximately 1628.15 USD in May 2023), and 67.1% of the participants did not have remunerated activities. Licit or illicit drugs were used by more than half of the participants (53.4%). Dentistry was the first choice of 67.8% of the students (Table 2).

**Table 2:** Frequency distribution of the sample and their mean grade among all finished oral surgery subjects

Variable		N (%) or Mean±SD	Mean - oral surgery subjects	p-value
Sex	Male	44 (30.1)	7.59 ± 0.64	0.082α
	Female	102 (69.9)	7.76 ± 0.52	
Age		23.39 ± 2.63	R=-0.284	<0.001&
Sexual orientation	Heterosexual	127 (87.0)	7.71 ± 0.56	0.972α
	Others	19 (13.0)	7.72 ± 0.57	
Monthly family income (in thousands R$)		8.20 ± 17.40	R=0.064	0.457&
Skin color	White	125 (85.6)	7.74 ± 0.57	0.159α
	Not-White	21 (14.4)	7.55 ± 0.50	
Dentistry as the first choice of graduation course	No	47 (32.2)	7.75 ± 0.61	0.530α
	Yes	99 (67.8)	7.69 ± 0.54	
Remunerated activities	No	98 (67.1)	7.67 ± 0.53	0.226α
	Yes	48 (32.9)	7.79 ± 0.62	
Licit or illicit drugs use during the course	No/Not used	68 (46.6)	7.76 ± 0.58	0.288α
	Yes	78 (53.4)	7.66 ± 0.55	

Legend: α t-test for independent samples; & Spearman correlation.

In the bivariable analysis, the sample showed that age had a statistically significant (p < 0.001) and inversely proportional association with academic performance (β: -0.284). In the final multivariable model, it was included the following variables: sex, age, remunerated activities, and drug use. The inversely proportional relationship between age and academic performance from the bivariable analysis was maintained in the multivariable analysis (p = 0.029). Regarding sex, women had better performance than men (p = 0.029). Concerning licit and illicit drug use, the students who used of drugs had worse academic performance (p = 0.048). Only remunerated activities did not show statistical significance related to academic performance (Table 3).

**Table 3:** Bi- and multivariable analysis, using linear regression, for the association between academic performance in surgery subjects and independent variables

	Bivariable		Multivariable	
	β **(95%CI)**	p-Value	β **(95%CI)**	p-Value
Sex		0.082		0.029
Masculine	Ref.		Ref.	
Feminine	0.144 (-0.203 - 0.375)		0.173 (0.022 - 0.400)	
Age	-0.284 (-0.094 - -0.027)	<0.001	-0.318 (-0.101 - -0.034)	0.029
Sexual orientation		0.972	-	-
Heterosexual	Ref.			
Others	0.036 (-0.269 - 0.279)			
Monthly family income (in thousands R$)	0.064 (-0.003 - 0.008)	0.457	-	-
Skin color		0.159	-	-
White	Ref.			
Not-White	-0.117 (-0.448 - 0.074)			
Dentistry as the first choice of graduation course		0.530	-	-
No	Ref.			
Yes	-0.052 (-0.260 - 0.134)			
Remunerated activities		0.226		0.079
No	Ref.		Ref.	
Yes	0.101 (-0.075 - 0.315)		0.139 (-0.020 - 0.351)	
Licit or illicit drugs use during the course		0.288		0.048
No/Not used	Ref.		Ref.	
Yes	-0.089 (-0.284 - 0.085)		-0.158 (-0.354 - -0.001)	

## DISCUSSION

The aim of this study was to evaluate the factors associated to academic performance in the field of oral surgery subjects among undergraduate dental students at a public university in southern Brazil. Our findings revealed significant associations between academic performance and three key factors: age, sex, and the use of licit or illicit drugs.

Academic performance is a multifaceted phenomenon influenced by various demographic, socioeconomic, psychological, and personal factors. Evaluating this intricate variable is crucial because it allows us to identify other variables that may either positively or negatively affect students' psychological well-being, healthcare provision to the population, and the future conduct of dentists ^1^^-^^9^.

In the current study, as students' age increased, their academic performance in oral surgery subjects tended to decline. These findings are consistent with a prior study that also demonstrated a decrease in academic performance as age increased ^3^. It may be speculated that less available time to study is involved in these findings. 

The current data indicated that women outperformed men in terms of academic performance. This finding aligns with previous research that has explored academic motivation in dental courses, revealing that women tend to exhibit higher levels of self-determination and motivation compared to men ^10^. Moreover, existing literature consistently points to a significant relationship between gender and grade point averages (GPA) among undergraduate dental students. Typically, a greater proportion of female students achieve higher GPAs compared to their male counterparts, reflecting differences in intrinsic and extrinsic goal orientations, strategies, and motivational factors between the two sexes.^11^^,^^12^

Women comprised roughly 70% of our sample, which may inflate crude mean differences, but should not bias adjusted estimates when sex is included in the adjusted model; even so, female sex remained a modest positive predictor of performance in the multivariable model (Table 2). Evidence in dental education is mixed across settings: a study from reported a significant association between sex and GPA among dental undergraduates ^11^. At the same time, other investigation suggested that apparent performance differences are better explained by motivational and self-regulatory factors than by sex per se. For instance, higher internal locus of control and self-efficacy ^13^, and high intrinsically motivated learning profiles, are linked to better academic outcomes independent of sex ^14^. Gender-linked patterns in diligence and achievement have also been described, with correlations varying by sex ^15^. Additionally, stress is a well-established determinant of dental students’ academic performance and may interact with gendered experiences of training ^6^. Taken together, while the female predominance in our sample may reduce the precision of estimates for males, the persistence of an adjusted female advantage suggests it is unlikely to be an artefact of sample imbalance, and may reflect underlying motivational and psychosocial differences. Moreover, the internal validity must be highlighted, as women represents the majority of dental students at the local institution. Further studies using more balanced samples and stratified analyses is warranted.

A noteworthy observation from the present study is that more than half of the participants reported using licit or illicit drugs, and among these students, those who had increased their drug use demonstrated poorer academic performance. These findings align with existing literature, which suggests a plausible link between substance abuse and its detrimental effects on learning among students in health sciences disciplines ^16^^-^^18^. Future research endeavors should aim to quantify the impact of substance use on dental student learning by employing objective assessment tools, moving beyond reliance solely on self-reported data. 

It is essential to consider that existing literature suggests that the impact on academic performance may vary relative to the workload, highlighting that it might not be employment itself that adversely affects academic outcomes, but rather the number of hours students devote to work while simultaneously attending the university ^4^. In essence, the amount and balance of work hours in relation to their study commitments may be a more critical factor in determining the impact on academic performance.

The existing literature underscores that both oral and maxillofacial surgeons and residents commonly experience high levels of occupational stress, which can potentially compromise their academic and professional performance-a phenomenon observed worldwide ^7^. While it may not be entirely equitable to draw direct comparisons, there is a discernible connection between residents and undergraduate students enrolled in oral surgery subjects, as suggested by previous studies ^5^^,^^6^^,^^19^.

Within oral surgery subjects, dental students frequently interact with patients who are not only in physical distress due to pain or possible traumatic injury, but are also dealing with aesthetic and functional issues resulting from dental extractions. Additionally, many of these patients harbor significant fears regarding the impending procedures. Collectively, these factors can significantly impact students' psychological well-being and academic performance, especially dental students facing their first oral surgery subject, when they are younger, with less experience, and more psychological pressure due to increased susceptibility to errors and inability to stress management ^7^. Consequently, it becomes evident that oral surgery subjects may exert a heightened level of stress compared to other subjects during the undergraduate course. Considering this, individuals since the first oral surgery subject completion were included. 

Ensuring a healthy learning environment and minimizing stressors can not only enhance students' quality of life but also contribute to the delivery of optimal healthcare to the population, aligning with previous research findings ^5^^-^^7^^,^^18^^,^^20^. To support dental students in managing stress, a multifaceted approach is essential. Regular monitoring of academic performance, including grades and retention rates, can help identify students at risk, enabling early intervention ^5^. Implementing stress management resources, such as mindfulness courses, coping skills workshops, and wellness counseling, can promote resilience and mental health ^20^. Collaborating with campus support services allows dental educators to address specific health issues that may impact academic progress ^7^. Furthermore, creating an inclusive and respectful learning environment that minimizes discrimination and harassment, coupled with healthy lifestyle guidance, can foster well-being ^18^. These efforts not only improve students’ academic outcomes but also may support their long-term success and contribution to the dental profession ^7^.

We also encourage further research to endeavor focus on comparing occupational stress among undergraduate dental students engaged in oral surgery subjects with those studying other subjects, with a keen eye on identifying the specific stressors unique to each. This could yield valuable insights into the nature of stress experienced by dental students in different academic contexts.

Among the limitations, it must be understood that academic performance is a complex outcome influenced by a multitude of factors, both objective and subjective, which can vary significantly among individuals. As this study was conducted in one university in Brazil, the generalizability of these results to broader contexts or diverse populations may be limited. Although the single institution data could be seen as a limiting factor, it also means that a higher internal validity can be expected, and thus a high external validity may be applied to other public Brazilian centers. Recognizing the multifaceted nature of academic performance and the uniqueness of individual experiences is crucial when considering the implications of this study. 

Despite these limitations, the study's design and its high response rate by the institution’s students allowed generalizing the data for comparisons with other studies assessing academic performance. Although this study is a secondary analysis with a smaller sample, the whole sample showed representativeness capacity with similar demographic data between the responders and non-responders students, and the analysis of variable associations, using an analytical epidemiological approach, holds significant importance for the development of healthcare management policies.

## CONCLUSIONS

This study revealed that academic performance in oral surgery subjects among undergraduate dental students was lower among older students and those who reported drug use. Conversely, female students demonstrated higher performance. Importantly, the presence of remunerated activities did not have a significant impact on academic performance.

## References

[1] McManus IC, Harborne AC, Horsfall HL, Joseph T, Smith DT, Marshall-Andon T (2020). Exploring UK medical school differences the MedDifs study of selection, teaching, student and F1 perceptions, postgraduate outcomes and fitness to practise. BMC Med.

[2] Dryer R, Henning MA, Tyson GA, Shaw R (2016). Academic achievement performance of university students with disability exploring the influence of non-academic factors. Int J Disabil Dev Edu.

[3] Ali S, Haider Z, Munir F, Khan H, Ahmed A (2013). Factors contributing to the students academic performance a case study of Islamia University Sub-Campus. Am J Edu Res.

[4] Rochford C, Connolly M, Drennan J (2009). Paid part-time employment and academic performance of undergraduate nursing students. Nurse Educ Today.

[5] Kernan WD (2019). Health-related impediments to learning among dental and oral surgery students. J Prev Interv Community.

[6] Elani HW, Allison PJ, Kumar RA, Mancini L, Lambrou A, Bedos C (2014). A systematic review of stress in dental students. J Dent Edu.

[7] Alkindi M, Alghamdi O, Alnofaie H, AlHammad Z, Badwelan M, Albarakati S (2020). Assessment of Occupational Stress Among Oral and Maxillofacial Surgeons and Residents in Saudi Arabia A Cross-Sectional Study. Adv Med Educ Pract.

[8] Fernandez MS, Pontes AFL, Casarin M, Feijo JS, Pola MN, Muniz FWMG (2023). Factors associated with poor academic performance among undergraduate dental students A cross-sectional study. J Dent Educ.

[9] Instituto Brasileiro de Geografia e Estatística (2008). Pesquisa das características étnicos-raciais da populacao..

[10] Santos AMC, Perazzo MF, Mattos FF, Pordeus IA, Granville-Garcia AF, Paiva SM (2022). Dental students’ satisfaction with their course and how it is associated to their satisfaction with life and career outlook. Acta Odontol Latinoam.

[11] Jowkar Z, Fattah Z, Asl ZK, Hamidi SA (2022). Stress, Sleep Quality, and Academic Performance among Dental Students in Shiraz, Iran. Int J Dent.

[12] Mafla AC, Divaris K, Herrera-López HM, Heft MW (2019). Self-Efficacy and Academic Performance in Colombian Dental Students. J Dent Educ.

[13] Ihm J-J, Lee G, Kim K-K, Jang K-T, Jin B-H (2013). Who succeeds at dental school? Factors predicting students’ academic performance in a dental school in republic of Korea. J Dent Educ..

[14] Orsini CA, Binnie VI, Tricio JA (2018). Motivational profiles and their relationships with basic psychological needs, academic performance, study strategies, self-esteem, and vitality in dental students in Chile. J Educ Eval Health Prof.

[15] Jedrychowski J, Lindemann R (2005). Comparing Standardized Measures of Diligence and Achievement with Dental Student Academic Performance. J Dent Educ.

[16] Al-Shatnawi SF, Perri MIII, Young HN, Norton M (2016). Substance use attitudes, behaviors, education and prevention in colleges of pharmacy in the United States. Am J Pharm Educ.

[17] Eckert PP, Finkelman M, Rosenberg MB (2016). Prevalence of substance abuse among oral and maxillofacial surgery residents from 2006 to 2015. J Oral Maxillofac Surg.

[18] Jackson ER, Shanafelt TD, Hasan O, Satele DV, Dyrbye LN (2016). Burnout and alcohol abuse/dependence among US medical students. Acad Med.

[19] Basudan S, Binanzan N, Alhassan A (2017). Depression, anxiety and stress in dental students. Int J Med Educ.

[20] Srivastava R, Jyoti B, Pradhan D, Kumar M, Priyadarshi P (2020). Evaluating the stress and its association with stressors among the dental undergraduate students of Kanpur city, India A cross-sectional study. J Edu Health Promot.

